# The Impact of Bariatric Surgery on Weight Loss and Glycemic Control in Patients With Obesity and Type 2 Diabetes: A Systematic Review

**DOI:** 10.7759/cureus.49122

**Published:** 2023-11-20

**Authors:** Hyder Mirghani, Sultan Abdulrahman S Alamrani, Amira A Alkonani, Abdullah M Al Madshush

**Affiliations:** 1 Internal Medicine, University of Tabuk, Tabuk, SAU; 2 Faculty of Medicine, University of Tabuk, Tabuk, SAU

**Keywords:** overweight, t2dm, glycemic control, weight loss, bariatric surgery

## Abstract

Obesity and type 2 diabetes mellitus (T2DM) are global health challenges. Bariatric surgery has emerged as a potential intervention for managing these conditions, but its efficacy and impact need comprehensive evaluation. This systematic review aimed to assess the impact of bariatric surgery on weight loss and glycemic control in patients with obesity and T2DM. Following Preferred Reporting Items for Systematic Reviews and Meta-Analyses (PRISMA) guidelines, a systematic search was conducted in October 2023, primarily using PubMed. Studies were selected based on specific inclusion and exclusion criteria, focusing on bariatric surgery's relationship with weight loss and glycemic control. The quality of the included studies was assessed using the ROBINS-I (risk of bias in non-randomized studies of interventions) risk of bias assessment approach. Out of 272 initially identified studies, nine met the inclusion criteria. These studies, encompassing 10,445 participants from various global locations, predominantly targeted middle-aged participants. The findings consistently highlighted the benefits of bariatric surgery in weight reduction and improved glycemic control. However, the degree of benefits varied based on the type of surgical procedure, patient's BMI, and other individual factors. Bariatric surgery offers significant advantages in managing obesity and T2DM. While it consistently aids in weight reduction and glycemic control, individualized treatment approaches considering various patient and procedural factors are crucial for optimal outcomes. When applied to the right patient, bariatric surgery can offer significantly better glycemic control and weight reduction when compared to only medication control and lifestyle adjustments. However, future research should focus on long-term outcomes and the integration of surgical interventions with lifestyle and medical management.

## Introduction and background

Obesity and type 2 diabetes (T2D) are two of the most formidable health challenges of the twenty-first century. The World Health Organization (WHO) estimates that more than 650 million adults were obese as of 2016, representing about 13% of the world's adult population [1]. Parallel to the rise in obesity, the global prevalence of diabetes has also surged. In 2019, approximately 463 million adults were living with diabetes, with type 2 diabetes accounting for 90% of these cases [2]. The convergence of these epidemics is no coincidence; obesity is a primary risk factor for the development of T2D, with approximately 90% of people living with T2D being overweight or obese [3].

While lifestyle modifications, including dietary changes and increased physical activity, are fundamental to managing both obesity and T2D, their effectiveness can be limited for many individuals, especially in the long term. As such, alternative therapeutic options have been sought. Bariatric surgery, initially conceptualized for weight reduction, has emerged as a powerful tool not only for weight loss but also for the remission of T2D. The American Diabetes Association, recognizing its potential benefits, has incorporated bariatric surgery into its clinical practice recommendations for select patients with obesity and T2D [4].

Several types of bariatric surgery exist, including Roux-en-Y gastric bypass (RYGB), sleeve gastrectomy, and adjustable gastric banding. Each procedure has its advantages, risks, and potential outcomes. Schauer et al. (2012) demonstrated that RYGB and sleeve gastrectomy led to significant improvements in glycemic control compared to medical therapy alone, with some patients achieving complete remission of T2D [5].

The mechanisms by which bariatric surgery improves glycemic control extend beyond weight loss alone. Notably, post-surgical hormonal changes, particularly the elevation of glucagon-like peptide-1 (GLP-1) and peptide YY, play a crucial role in enhancing insulin secretion and sensitivity [6]. Additionally, alterations in gut microbiota post-surgery have also been proposed to contribute to improved metabolic profiles [7].

However, while the potential benefits of bariatric surgery are promising, they must be weighed against the risks. Complications such as nutritional deficiencies, dumping syndrome, and surgical complications, though relatively rare, are pertinent considerations for prospective candidates [8]. Moreover, the long-term durability of diabetes remission after bariatric surgery remains an area of ongoing research.

In this exploration of the impact of bariatric surgery on weight loss and glycemic control, we will delve deep into the available scientific literature, scrutinize clinical trials, and analyze patient outcomes. The overarching objective is to paint a comprehensive picture, elucidating the potential benefits and challenges of bariatric surgery for individuals with obesity and type 2 diabetes.

## Review

Methods

Study Design and Duration

This was a systematic review conducted in October 2023. Preferred Reporting Items for Systematic Reviews and Meta-Analyses (PRISMA) guidelines were followed for this systematic review.

Search Strategy

To retrieve the relevant research, a thorough search was conducted across major databases, using PubMed mainly as a search engine for studies. We only searched in English. The following keywords were converted into PubMed Mesh terms and used to find studies that were related; "bariatric," "surgery," “weight,” “loss” "glycemic," "control," “patients,” “obesity,” “type 2” and "diabetes”." The Boolean operators "OR" and "AND" matched the required keywords. Among the search results were publications in full English language, freely available articles, and human trials.

We considered the following criteria for inclusion in this review: Studies that investigated the relationship between bariatric surgery, weight loss, and glycemic control; clinical trials; observational studies; controlled clinical trials; comparative studies; and free-access articles.

The exclusion criteria were: Systemic reviews; studies that focused on type 1 diabetes; article reviews; meta-analyses; studies earlier than 2015; case reports, letters to the editors, and replies to conflicts; and non-English language.

Data Extraction

Duplicates in the search strategy output were found using Rayyan (QCRI) [9]. To determine the relevance of titles and abstracts, the researchers used a set of inclusion/exclusion criteria to filter the combined search results. The reviewers carefully read each paper that matched the requirements for inclusion. The authors provided other methods of resolving disputes with some thought. The authors extracted data about the study titles, authors, study year, country, participants, gender, diagnostic tool, main outcomes, and conclusion.

Strategy for Data Synthesis

Summary tables were created using information from pertinent research to give a qualitative overview of the results and study components. Following data extraction for the systematic review, the most effective strategy for utilizing data from the included study articles was selected.

Risk of Bias Assessment

Using the ROBINS-I (risk of bias in non-randomized studies of interventions) risk of bias assessment approach for non-randomized trials of therapies, the included studies' quality was assessed [10]. The seven themes that were assessed were confounding, participant selection for the study, classification of interventions, deviations from intended interventions, missing data, assessment of outcomes, and choosing of the reported result.

Results

**Figure 1:**
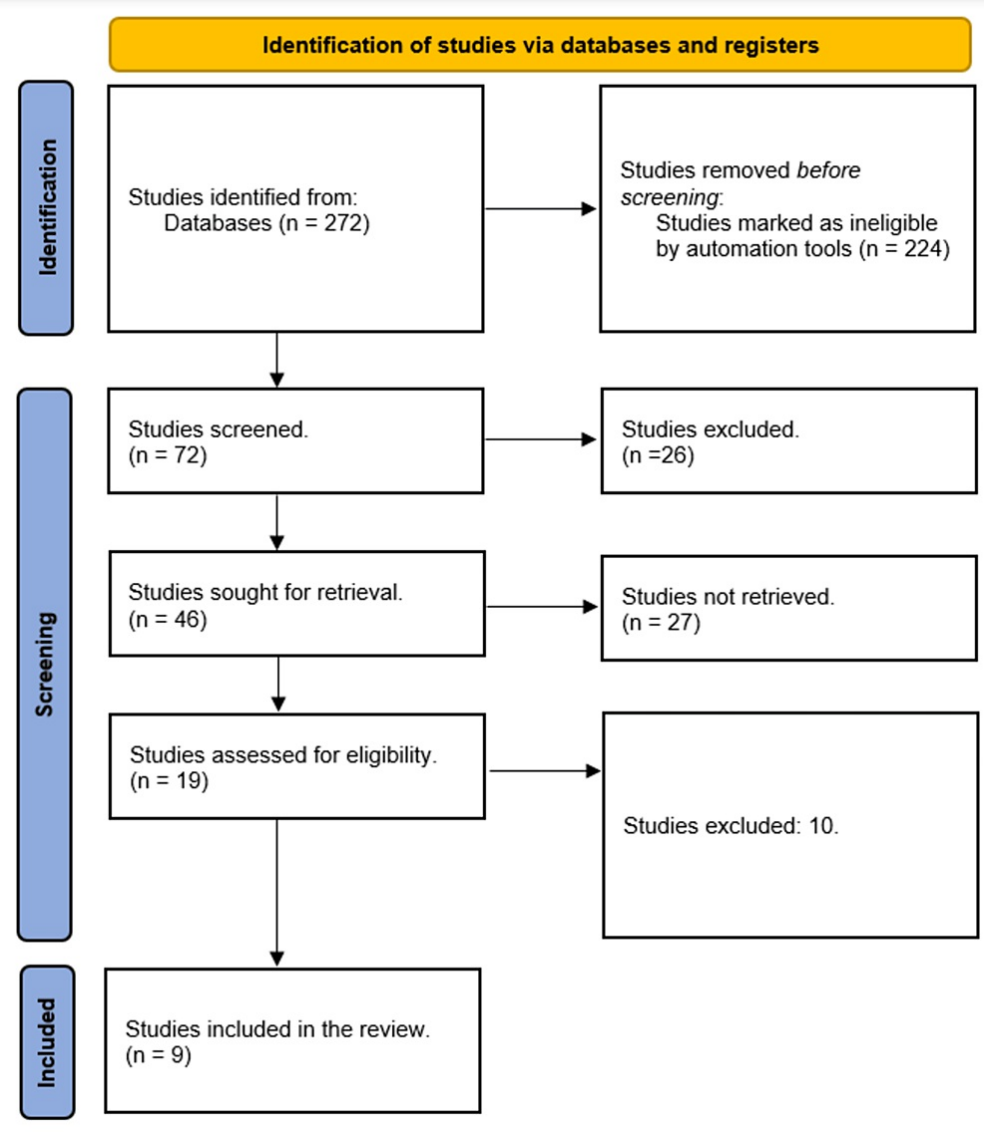
PRISMA flowchart summarizes the study selection process PRISMA: Preferred Reporting Items for Systematic Reviews and Meta-Analyses

A total of 272 study articles resulted from the systematic search, and 224 were automatically removed. Title and abstract screening were conducted on 72 studies, and 26 studies were excluded. Forty-six studies were sought for retrieval, and only 19 articles were retrieved. Finally, 19 studies were screened for full-text assessment; 10 studies were excluded for either having inappropriate study methodology or results. Nine eligible study articles were included in this systematic review. A summary of the study selection process is presented in Figure 1.

Sociodemographic Characteristics of Participants in Recent Studies

Table 1 delineates the sociodemographic specifics of the 10,445 participants from nine different studies [11-19] encompassing a diverse range of study designs and locations. The participant count from the studies was quite varied, with McTigue KM et al. (2020) leading the list, having researched a substantial cohort of 9710 participants [14]. The studies are geographically varied, covering countries and regions such as the USA, Europe, Japan, China, Brazil, and Taiwan. In terms of male representation, Ding SA et al. (2015) [11] reported a male percentage of 55%. On the other hand, McTigue KM et al. (2020) [14] noted a significantly lower male percentage, standing at 27.4%. Wang X et al. (2016) [15] recorded a male participation of 42.8%, and Nguyen KT et al. (2015) [18] noted a near-equal representation of 48.5% of males. Notably, several studies like Wallenius V et al. (2015) [12], Seki Y et al. (2022) [13], Coelho D et al. (2018) [16], Ikramuddin S et al. (2016) [17], and Petry TZ et al. (2015) [19] did not specify the gender ratio. Study designs varied, with some following a prospective approach, some being retrospective, while others were randomized clinical trials or cohort studies. As for the age data, where provided, the age range seemed to majorly cater to middle-aged participants. Specifically, Ding SA et al. (2015) [11] reported an age mean of 51 ± 10 years, Seki Y et al. (2022) [13] detailed an average age of 47.8 ± 7.7 years, McTigue KM et al. (2020) [14] indicated a mean age of 49.8 years, Wang X et al. (2016) [15] observed a significantly younger average age of 30.33±8.61 years, and Nguyen KT et al. (2015) [18] showed a mean age of 50 ± 1 years. To sum up, the studies showcased in Table 1 predominantly focus on middle-aged participants, even though the participant count and locations vary considerably.

**Table 1:** Sociodemographic characteristics of the included participants

Study	Study design	Location	Participants	Age range (mean) in years	Males (%)
Ding SA, et al. 2015. [11]	Prospective, randomized clinical trial.	USA	45	51 ± 10	55%
Wallenius V, et al. 2015. [12]	Prospective clinical Trial	Multicenter (Europe)	33	-	-
Seki Y, et al. 2022. [13]	A retrospective study.	Japan	316	47.8 ± 7.7	-
McTigue KM, et al. 2020. [14]	Cohort study	USA	9710	49.8	27.4%
Wang X, et al. 2016. [15]	Retrospective analysis	China	70	30.33±8.61	42.8%
Coelho D, et al. 2018. [16]	A Comparative study	Brazil	66	-	-
Ikramuddin S, et al. 2016. [17]	Randomized controlled trial.	USA and Taiwan	120	-	-
Nguyen KT, et al. 2015. [18]	Ancillary study	USA	68	50 ± 1	48.5%
Petry TZ, et al. 2015. [19]	Randomized Controlled Trial	Brazil	17	47±8	-

Table 2 highlights various studies examining the clinical characteristics and outcomes of different surgical and non-surgical treatments for type 2 diabetes (T2D) and obesity. Ding SA et al. [11] compared the effects of laparoscopic adjustable gastric band (LAGB) with an intensive medical diabetes and weight management (IMWM) program. The study found both treatments comparable in terms of diabetes control, cardiometabolic risk, and patient satisfaction over a year, even though there were differences in weight loss and systolic blood pressure reduction. Two studies, Wallenius V et al. [12] and McTigue KM et al. [14], delved into the glycemic effects of Roux-en-Y gastric bypass (RYGB) and laparoscopic sleeve gastrectomy (LSG) on obese T2D patients. While both treatments showed similar glycemic control outcomes, RYGB patients had superior results in terms of weight loss, diabetes remission, relapse rate, and long-term glycemic control compared to LSG patients. Seki Y et al. [13] focused on the Japanese population, comparing bariatric surgery with medical treatment for mildly obese T2D patients. Bariatric surgery proved to be more clinically and cost-effective, with a striking difference in diabetes remission rates and drug costs between the two groups. Wang X et al. [15] assessed the effectiveness of LSG in treating obesity in Chinese patients. The surgery was found to be notably effective for weight loss and improving glycemic control, especially for patients with higher BMI. Two studies, Coelho D et al. [16] and Ikramuddin S et al. [17], explored the long-term impacts of RYGB. Both studies highlighted the efficacy of RYGB in achieving diabetes treatment goals, with the latter also pointing out the risks associated with the procedure over time.

**Table 2:** Objectives, results, and clinical outcomes of the included studies Hemoglobin A1C (HbA1c), Body Mass Index (BMI), Total Weight Loss (TWL), Excess Weight Loss (EWL), Standard Deviation (SD), Type 2 Diabetes Mellitus (T2DM), Bariatric Surgery (BS), Roux-en-Y Gastric Bypass (RYGB), Laparoscopic Sleeve Gastrectomy (LSG), Laparoscopic Roux-en-Y Gastric Bypass (LRYGB), Sleeve Gastrectomy (SG), Duodenal-Jejunal Bypass Surgery with Minimal Gastric Resection (DJBm)

Study	Objective	Results	Outcomes
Ding SA, et al. 2015. [11]	The study aimed to compare the effects of a laparoscopic adjustable gastric band (LAGB) to an intensive medical diabetes and weight management (IMWM) program for type 2 diabetes (T2D) treatment.	Out of the 40 participants who began the intervention, 33% in the LAGB group achieved the primary glycemic endpoint, compared to 23% in the IMWM group. Both groups saw similar reductions in HbA1c levels at 3 and 12 months. However, weight loss was more significant in the LAGB group after 12 months. Systolic blood pressure decreased more in the IMWM group, while other metrics like diastolic blood pressure, lipids, and fitness saw comparable changes between the two groups.	Both LAGB and IMWM programs provided comparable benefits in terms of diabetes control, cardiometabolic risk, and patient satisfaction over a year. The choice between these treatments should consider various factors, including personal preference.
Wallenius V, et al. 2015. [12]	The study aimed to compare the glycemic effects of LRYGB (Roux-en-Y gastric bypass) and LSG (sleeve gastrectomy) in obese type 2 diabetic patients.	Both LRYGB and LSG resulted in decreased postoperative fasting blood glucose. GLP-1 levels were higher in LRYGB patients at 3 weeks post-operation. LRYGB showed a greater decrease in BMI than LSG.	LRYGB and LSG have similar effects on glycemic control, even though LSG has lower GLP-1 levels and a lesser decrease in BMI.
Seki Y, et al. 2022. [13]	The study aimed to compare the glycemic control of mildly obese patients with type 2 diabetes mellitus one year post-treatment between those who underwent bariatric surgery and those who received medical treatment in Japan.	59.0% of the BS group achieved diabetes remission (HbA1c <6.5% without medication) at 1 year compared to 0.4% in the MT group. The monthly drug costs for the metabolic syndrome in the BS group decreased from $US126.5 to $US0.0, whereas it increased from $US52.4 to $US58.3 in the MT group.	Bariatric surgery for mildly obese patients with type 2 diabetes mellitus is more clinically and cost-effective than medical treatment in Japan.
McTigue KM, et al. 2020. [14]	The study aimed to evaluate the associations of bariatric surgery with T2DM outcomes. Roux-en-Y gastric bypass (RYGB) vs sleeve gastrectomy (SG).	Of the total participants, RYGB patients experienced more weight loss and had a 10% higher T2DM remission rate and lesser T2DM relapse compared to SG patients. After 5 years, hemoglobin A1c reduced more in RYGB patients than in SG patients.	Patients who underwent RYGB had superior results in terms of weight loss, T2DM remission rate, relapse rate, and long-term glycemic control than those who had SG
Wang X, et al. 2016. [15]	The study aimed to assess the effectiveness and safety of Laparoscopic sleeve gastrectomy (LSG) in treating obesity in Mainland Chinese patients.	Seventy patients underwent LSG, and the primary outcome was total weight loss (%TWL) and excess weight loss (%EWL). At the end of three years, the %TWL was 33.7±7.1, and %EWL was 77.2±13.1. Glycemic control improved, with 69.2% achieving optimal levels at three years.	LSG is effective for weight loss and improving glycemic control in Mainland Chinese patients, especially those with a BMI of 40 kg/m2 or above.
Coelho D, et al. 2018. [16]	The study aimed to compare glycemic control after LRYGB between diabetic patients with BMI 30-35 kg/m^2 and those with BMI >35 kg/m^2, along with evaluating weight loss, comorbidities, and surgical morbidity.	Over an average follow-up of 4.3 years, there was no significant difference in complete diabetes remission between the groups. However, greater partial remission and lower levels of HbA1c were observed in the BMI >35 kg/m^2 group. Weight control was better in the BMI 30-35 kg/m^2 group.	The LRYGB procedure showed no significant difference in complete diabetes remission between the two BMI groups. However, the more obese group (BMI >35 kg/m^2) exhibited a better response in terms of partial diabetes remission and lower HbA1c levels.
Ikramuddin S, et al. 2016. [17]	The study aimed to compare the 3-year achievement of a composite treatment goal for diabetes after intensive lifestyle-medical management intervention with and without Roux-en-Y gastric bypass.	At 36 months, 9% of the lifestyle-medical management patients and 28% of the gastric bypass patients met the triple endpoint goal. Gastric bypass patients demonstrated improved HbA1c values and greater weight loss and used fewer medications compared to the lifestyle-medical management group.	Gastric bypass was more effective in achieving diabetes treatment goals, primarily due to better glycemic control. However, its effect decreased over time and was linked to more adverse events.
Nguyen KT, et al. 2015. [18]	This study investigated changes in incretins, insulin sensitivity, and secretion 1 year after patients underwent lifestyle modification and intensive medical management (LS/IMM) alone or in combination with Roux-en-Y gastric bypass (RYGB).	The RYGB group showed more weight loss and a significant improvement in HbA1c than the LS/IMM group. Glucagon-like peptide 1 (GLP-1) increased notably after RYGB, while other hormones decreased. Weight loss was the major factor correlating with reduced HbA1c in both groups.	Weight loss and retained β-cell function mainly determine the improved glycemic benefit after RYGB.
Petry TZ, et al. 2015. [19]	The study aimed to assess if the upper gastrointestinal tract (UGI) bypass impacts glucose regulation factors in type 2 diabetes (T2D) patients.	DJBm (duodenal-jejunal bypass) surgery led to weight loss and improvements in metabolic function. After surgery, subjects experienced better glucose tolerance, improved insulin sensitivity, an enhanced insulin response to glucose, and better overall glycemic control compared to those receiving standard care.	DJBm promotes moderate weight loss and metabolic function improvements in T2D patients. However, it's unclear if the benefits arise from weight loss or the UGI tract bypass itself.

Nguyen KT et al. [18] investigated the impact of lifestyle modification combined with RYGB. The combined approach showed better weight loss and improved HbA1c, with weight loss playing a pivotal role in improved glycemic benefit post-surgery. Lastly, Petry TZ et al. [19] analyzed the effects of upper gastrointestinal tract (UGI) bypass on glucose regulation in T2D patients. The surgery, dubbed duodenal-jejunal bypass (DJBm), demonstrated benefits in weight loss and metabolic function. However, the precise reason for these benefits remains uncertain, whether they are due to weight loss or the UGI tract bypass itself. In essence, the studies underscore the effectiveness of various surgical procedures in managing T2D and obesity. While the outcomes differ in specifics, the overarching consensus is the prominence of weight loss as a significant factor influencing glycemic control and metabolic improvements.

Ding et al. (2015) [11] examined the outcomes of laparoscopic adjustable gastric banding, reporting a significant 12-month weight loss of -13.5 ± 1.7 kg. Additionally, they noted that 33% of the subjects achieved the target HbA1c level below 6.5% at the end of the 12-month period. Wallenius et al. (2015) [12] compared the effects of laparoscopic Roux-en-Y gastric bypass (LRYGB) and laparoscopic sleeve gastrectomy (LSG). They found that the BMI decreased more after LRYGB than LSG, with fasting blood glucose levels showing similar reductions in both procedures at 12 months. Seki et al. (2022) [13] investigated the impact of bariatric surgery on weight loss and diabetes remission. They reported a mean decrease in BMI of -7.5 ± 2.8 kg/m^2^ at one year, with 59.0% of the bariatric surgery group achieving diabetes remission (HbA1c <6.5%) at the same time point. The mean change in HbA1c was -1.6 ± 1.2%. McTigue et al. (2020) [14] compared the outcomes of RYGB and sleeve gastrectomy (SG) in terms of weight loss and type 2 diabetes mellitus (T2DM) remission. They found that both procedures led to substantial weight loss at one year, with T2DM remission rates of 59.2% for RYGB and 55.9% for SG. Wang et al. (2016) [15] focused on laparoscopic sleeve gastrectomy (LSG) and reported a total weight loss percentage (%TWL) of 33.7±7.1 and an excess weight loss (%EWL) of 77.2±13.1 at the end of three years. They also noted significant improvements in glycemic control, with 69.2% of the subjects achieving optimal levels at three years. Coelho et al. (2018) [16] studied the effects of LRYGB and reported a %TWL of 20.7%, along with an average reduction in HbA1c levels over time of -1.26%. Ikramuddin et al. (2016) [17] investigated the outcomes of RYGB and found a mean weight loss of 21.0% (SD 14.5) and a significant decrease in HbA1c values at three years. Nguyen et al. (2015) [18] reported a weight loss of 28.1 ± 1.3% after one year following RYGB, with HbA1c levels showing a substantial reduction from 9.8 ± 0.2 to 6.4 ± 0.2 after 12 months. Petry et al. (2015) [19] investigated the effects of DJBm and found a 10.2 ± 4.3% decrease in body weight after one year.

Table 3 lists the changes in weight loss and glycemic control in the included studies.

**Table 3:** Weight loss and glycemic control change in the included studies Hemoglobin A1C (HbA1c), Body Mass Index (BMI), Total Weight Loss (TWL), Excess Weight Loss (EWL), Standard Deviation (SD), Type 2 Diabetes Mellitus (T2DM), Bariatric Surgery (BS), Roux-en-Y Gastric Bypass (RYGB), Laparoscopic Sleeve Gastrectomy (LSG), Laparoscopic Roux-en-Y Gastric Bypass (LRYGB), Sleeve Gastrectomy (SG), Duodenal-jejunal Bypass Surgery with Minimal Gastric Resection (DJBm)

Study	Procedure(s)	Weight Loss	Glycemic Control
Ding SA, et al. 2015. [11]	Laparoscopic adjustable gastric band	12-month weight loss of −13.5 ± 1.7 kg	Six of 18 subjects (33% reached the target HbA1c level below 6.5% at 12 months
Wallenius V, et al. 2015. [12]	LRYGB (Roux-en-Y gastric bypass) and LSG (sleeve gastrectomy.	(BMI) decreased more after LRYGB than LSG (− 10.1 ± 0.9 vs. − 7.9 ± 0.5 kg/m2)	fasting blood glucose decreased Similarly (LRYGB vs. SG; 12 months —6.6 ± 0.4 vs. 5.9 ± 0.4)
Seki Y, et al. 2022. [13]	Bariatric surgery	Mean changes in BMI were −7.5 ± 2.8 kg/m2 at 1 year	59.0% of the BS group achieved diabetes remission (HbA1c <6.5%) at 1 year. Mean changes in HbA1c Was −1.6 ± 1.2%.
McTigue KM, et al. 2020. [14]	Roux-en-Y gastric bypass (RYGB) vs sleeve gastrectomy (SG).	weight loss 1 year after surgery (SG, −22.8% RYGB, −29.1%)	T2DM remission for RYGB and SG were 59.2% vs 55.9%, at 1 year
Wang X, et al. 2016. [15]	Laparoscopic sleeve gastrectomy (LSG)	At the end of 3 years, the %TWL (total weight loss) was 33.7±7.1, and %EWL (excess weight loss) was 77.2±13.1.	Glycemic control improved, with 69.2% achieving optimal levels at 3 years.
Coelho D, et al. 2018. [16]	Laparoscopic Roux-En-Y Gastric Bypass (LRYGB)	TWL% were 20.7%	HbA1c levels over time were on average -1.26%
Ikramuddin S, et al. 2016. [17]	Roux-en-Y gastric bypass (RYGB).	% weight loss mean was (SD) was 21.0% (14.5)	Mean (SD) HbA1c values at 3 years were 6.7% (2.0) (P < 0.001)
Nguyen KT, et al. 2015. [18]	Roux-en-Y gastric bypass (RYGB).	% weight loss was 28.1 ± 1.3 after 1-year	HbA1c (%) before and after 12 months were 9.8 ± 0.2 and 6.4 ± 0.2.
Petry TZ, et al. 2015. [19]	Duodenal-jejunal bypass surgery with minimal gastric resection (DJBm)	Body weight decreased 10.2 ± 4.3% after 1 year	Body weight before and after 12 months were 85±13 and 77±12.

Discussion

Obesity and type 2 diabetes are two closely related conditions. Obesity is a major risk factor for developing type 2 diabetes, and people with type 2 diabetes are often obese. Both conditions are associated with a range of health problems, including cardiovascular disease, stroke, and kidney disease. It is therefore important to find effective treatments for these conditions [2]. Bariatric surgery is a treatment option that has been shown to be effective in helping patients with obesity and type 2 diabetes. The surgery involves reducing the size of the stomach, which helps limit the amount of food that can be eaten. This, in turn, leads to weight loss. In addition to weight loss, bariatric surgery has been shown to have a positive impact on glycemic control in patients with type 2 diabetes [4]. 

Several studies have been conducted to evaluate the impact of bariatric surgery on weight loss and glycemic control in patients with obesity and type 2 diabetes. One study found that patients who underwent bariatric surgery had a significant reduction in body weight and an improvement in glycemic control compared to those who did not undergo surgery. Another study found that bariatric surgery was associated with a reduction in the need for diabetes medication [9]. The benefits of bariatric surgery on weight loss and glycemic control in patients with obesity and type 2 diabetes are clear. However, it is important to note that the surgery is not without risks. Patients who undergo bariatric surgery may experience complications such as bleeding, infection, and bowel obstruction. It is therefore important for patients to carefully consider the risks and benefits of the surgery before making a decision [6]. Bariatric surgery has been shown to have a significant impact on weight loss and glycemic control in patients with obesity and type 2 diabetes. This procedure is an effective treatment option for these conditions, but it is important for patients to carefully consider the risks and benefits before making a decision. Healthcare professionals should also be aware of the benefits and risks of bariatric surgery and be able to provide patients with accurate and up-to-date information to help them make informed decisions about their treatment options [7]. The studies presented offer a comprehensive insight into various surgical and medical interventions for managing obesity and type 2 diabetes mellitus (T2DM). A common thread among these studies is the evaluation of the efficacy of different bariatric surgical procedures compared to medical or lifestyle interventions.

The sociodemographic details of 10,445 participants from nine diverse studies [11-16], spanning various designs and global locations. While McTigue KM et al. (2020) [14] had the largest cohort, with 9,710 participants, the studies collectively covered regions like the USA, Europe, Japan, China, Brazil, and Taiwan. The male representation varied, with Ding SA et al. (2015) [11] having 55% males and McTigue KM et al. (2020) [14] only 27.4%. Several studies didn't specify gender distribution. The majority of these studies targeted middle-aged participants, with ages predominantly ranging around the late 40s to early 50s. Overall, the studies offer a comprehensive view of diverse participants across different geographies and study designs.

Ding SA et al. [11] and Wallenius V et al. [12] both focused on comparing different bariatric surgical methods. Ding SA et al. [11] compared the laparoscopic adjustable gastric band (LAGB) with the intensive medical diabetes and weight management (IMWM) program. Over a year, both treatments provided comparable benefits in terms of diabetes control, cardiometabolic risk factors, and patient-reported satisfaction. This suggests that the choice between these treatments should be based on individual patient preference and other factors beyond the study's findings. On the other hand, Wallenius V et al. [12] compared the early weight-independent and later weight-dependent glycemic effects of LRYGB and LSG in obese T2DM patients. Both procedures showed similar effects on glycemic control, but LSG had lower GLP-1 levels and a less pronounced reduction in BMI compared to LRYGB.

Seki Y et al. [13] provided a unique perspective by comparing bariatric surgery with standard medical treatments in Japan. Their findings highlighted that bariatric surgery is both clinically superior and more cost-effective than standard medical treatments for mildly obese T2DM patients. This is evident from the higher remission rates, significant improvements in metabolic parameters, and substantial reductions in associated drug costs for those who underwent surgery. McTigue KM et al. [14] evaluated the benefits and outcomes of two types of bariatric surgeries, RYGB and SG, in association with T2DM outcomes. Their multicenter study across the US found that patients undergoing RYGB had better outcomes in terms of weight loss, T2DM remission and relapse rates, and glycemic control compared to those undergoing SG. These results can guide surgical decisions for individuals considering bariatric surgery for T2DM management.

Lastly, Wang X et al. [15] focused on the efficiency and safety of LSG for Mainland Chinese patients. Their study found that LSG has shown significant effectiveness in weight reduction and control of blood glucose levels, especially beneficial for those with a higher BMI (≥40 kg/m^2^). Coelho D et al. [16] explored the efficacy of the LRYGB procedure in two distinct BMI groups. Interestingly, while both groups witnessed significant metabolic improvements, the group with a BMI greater than 35 kg/m^2^ exhibited a more robust response in terms of partial diabetes remission and improved HbA1c levels. This suggests that while LRYGB is effective across different BMI categories, the degree of obesity might influence the extent of metabolic benefits post-surgery. Ikramuddin S et al. [17] provided a comparative analysis between lifestyle-medical management and Roux-en-Y gastric bypass. The study underscored the superior efficacy of the gastric bypass in achieving diabetes treatment objectives, particularly in glycemic control. However, it's noteworthy that the benefits of the surgery seemed to diminish over time, and it was associated with a higher incidence of adverse events. This highlights the need for a comprehensive evaluation of the long-term risks and benefits of bariatric surgery versus lifestyle-medical management.

Nguyen KT et al. [18] delved into the underlying mechanisms responsible for improvements in T2DM post-RYGB. Their findings emphasize that weight loss, coupled with preserved β-cell functionality, is pivotal in driving the enhanced glycemic benefits post-surgery. This study provides valuable insights into the multifaceted mechanisms through which RYGB exerts its therapeutic effects, beyond mere weight loss. Lastly, Petry TZ et al. [19] investigated the potential benefits of bypassing the upper gastrointestinal tract (UGI) in T2DM patients. The study found that the DJBm surgical procedure led to moderate weight loss and improved metabolic function. However, it remains inconclusive whether the observed benefits are solely attributed to weight loss or if the UGI bypass itself has intrinsic therapeutic properties.

## Conclusions

The included studies collectively emphasize the multifactorial benefits of bariatric surgical interventions in T2DM management. They highlight the importance of individualized treatment approaches, considering the patient's specific BMI, the type of surgical procedure, and the potential long-term outcomes. The collective evidence underscores the need for a holistic approach in T2DM management, integrating surgical interventions with lifestyle and medical management, to achieve optimal patient outcomes.
